# Identifying main finding sentences in clinical case reports

**DOI:** 10.1093/database/baaa041

**Published:** 2020-06-11

**Authors:** Mengqi Luo, Aaron M Cohen, Sidharth Addepalli, Neil R Smalheiser

**Affiliations:** 1 Department of Psychiatry and Psychiatric Institute, University of Illinois College of Medicine, Chicago, IL 60612, USA; 2 School of Information Management, Wuhan University, Wuhan, Hubei 430072, China; 3 Department of Medical Informatics and Clinical Epidemiology, Oregon Health & Science University, Portland, OR 97239, USA

## Abstract

Clinical case reports are the ‘eyewitness reports’ of medicine and provide a valuable, unique, albeit noisy and underutilized type of evidence. Generally, a case report has a single main finding that represents the reason for writing up the report in the first place. However, no one has previously created an automatic way of identifying main finding sentences in case reports. We previously created a manual corpus of main finding sentences extracted from the abstracts and full text of clinical case reports. Here, we have utilized the corpus to create a machine learning-based model that automatically predicts which sentence(s) from abstracts state the main finding. The model has been evaluated on a separate manual corpus of clinical case reports and found to have good performance. This is a step toward setting up a retrieval system in which, given one case report, one can find other case reports that report the same or very similar main findings. The code and necessary files to run the main finding model can be downloaded from https://github.com/qi29/main_ finding_recognition, released under the Apache License, Version 2.0.

## Introduction

Clinical case reports are an interesting test bed for text mining, information extraction research and evidence-based medicine (1–4). One of their main drawbacks is they represent a type of evidence that is ‘noisy’ and uncontrolled (e.g. no placebo controls or randomization in place); therefore, one would place more confidence in a finding that is reported multiple times in the literature (2) rather than once. To ‘add up’ evidence across case reports (6–8) requires having a means to identify the case reports that have the most similar findings.

Generally, a clinical case report has a single main finding that represents the reason for writing up the report in the first place. Our previous studies have shown that the main finding of a clinical case report is almost always stated in the title and repeated in one or two sentences within the abstract (5). This is a much simpler situation than is encountered in other types of articles such as clinical trials, preclinical animal studies or biochemical experiments, where identifying statements of knowledge claims requires more advanced linguistic analysis and argumentation mining (9, 10). If the title already expresses the main finding, one may ask why we still seek to identify the main finding sentence within the abstract. There are several reasons for this. Identifying main finding sentences is necessary for the following goals. (i) In our preliminary studies, we have found that the title alone does not provide sufficient information to index a case report article according to main finding. Thus, we hypothesize that combining text judiciously from both title and main finding sentence(s) will provide additional information that will assist in indexing. (ii) As well, characterizing the similarity between the title and main finding sentence of the same article should allow us to create a similarity metric that can be applied to identify similar main findings across different articles as well. (iii) Identifying main finding sentences of case reports automatically at scale should provide a large corpus for a deeper analysis of rhetorical features that can be generalized for tackling the more difficult situations of identifying main finding sentences within other types of articles, e.g. clinical trial articles. (iv) Finally, the main finding is often repeated with variations in the Introduction and Summary or Conclusions sections of full text (2); the multiple different statements of the same main finding, in the same paper, should provide a novel textual resource for studying paraphrases and natural language inference.

In our previous study, we created a manual corpus of main finding sentences extracted from the abstracts and portions of full text of clinical case reports (5). Here, we have utilized the corpus to create a machine learning-based model that automatically predicts which sentence(s) from the abstract state the main finding. The performance of the model has been evaluated on a separate manual corpus of main finding sentences. The software is open and available on Github [https://github.com/qi29/main_finding_recognition].

## Materials and Methods

A sentence (or title) is said to state a main finding if it expresses the novel, surprising or interesting finding that motivated the authors to write up the case report for publication. For example, ‘This, to our knowledge, is the first report of a nose growing out of a person’s ear.’ Note that the sentence that states the main finding is generally NOT the same as the sentence that states the ‘take home message’—the latter provides context for the main finding, elaborates on it, asserts its importance or points out implications for clinicians. Nor is the patient presentation generally the main finding (e.g. ‘We saw a 3-year old female who presented with fever and rash.’).

**Figure 1:**
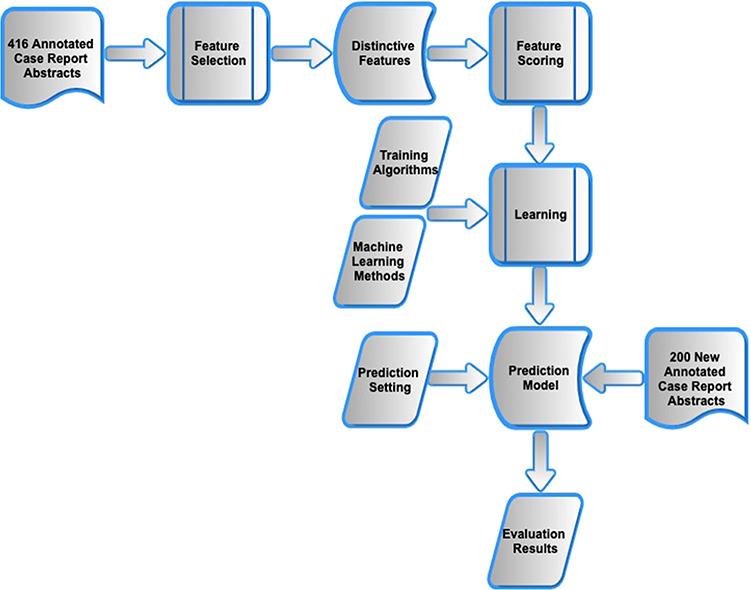
Workflow of methods.

As shown in Figure 1, the methods progressed through three stages. First, using the previously presented manual corpus of 416 annotated case reports (5), feature selection and encoding was carried out and applied to each sentence within an abstract. Second, different machine learning models were investigated that combined the features into a composite score and were trained to distinguish manually annotated main finding sentences from all other sentences within the abstracts of the same case reports. Third, the optimal model was evaluated on a new manual corpus of 200 clinical case reports.

### Feature selection and encoding

The previously reported manual corpus consists of 416 titles, main finding sentences (annotated with agreement by both annotators from abstracts), 55 alternative main finding sentences (a second sentence in the abstract that was identified by at least one annotator), and 2985 other sentences (5). Abstract text was split into sentences using the Natural Language Toolkit (NLTK) (version 3.4) package in Python 2.7 for sentence tokenization and word tokenization. All words were handled without stemming or lemmatization.

For machine learning, we employed feature selection, feature engineering and feature combination in a supervised learning framework (11). Main finding sentences and alternative main finding sentences were both regarded as positive examples, and all other sentences were regarded as negative examples. (Note that some of the sentences not annotated might arguably be statements of the main finding, at least in part.)

To identify features that distinguish positive sentences vs. negative sentences based on statistical criteria, we extracted, scored and combined five features as discussed in the following sections.

#### Features 1 and 2: similarity between sentence and title

In over 90% of cases, the title of a case report states its main finding in a concise fashion (5). Hence, if a sentence within the abstract also states the main finding, we expected that it would show textual or semantic similarity with the title.

##### Feature 1. n-gram-based similarity

We compared the title with each sentence in the abstract, according to how many word n-grams they shared. For instance, if the same sequence of eight words is expressed in both title and sentence, we assign a score of 8*8 to that sentence. That 8-gram is removed and we then compare 7-grams, 6-grams and so on down to single words (1-grams). A stop word list consisting of the about 1400 most frequent words in PubMed titles and abstracts (Table S1) was applied before comparing single words. For n-gram score calculation over all n-grams, we use the following formula

(1) \begin{align*} \mathrm{Similarity}\ \mathrm{Score}=\sum_0^nn\ast n, \end{align*}

where n ranges between 1 and 8. For example, for the title ‘The effects of injection of bovine vaccine into a human digit: a case report.’ and a sentence ‘We report accidental injection of bovine vaccine into the base of the little finger.’, there is only a 5-gram that appears in both title and sentence, so the score of this sentence is 5^*^5 = 25.

##### Feature 2. Semantic similarity

As the n-gram-based similarity method only considers exact sequences of word matches, it cannot deal with changing the order of words or the use of word variants or synonyms. In order to compare the semantic similarity in title vs. each sentence in the abstract, we applied the biomedical sentence embeddings model provided in (12) to represent each sentence and the title as vectors after applying a stop word list (Table S1), and then the cosine similarity was calculated between the two vectors.

#### Feature 3. Frequent textual patterns found in main finding sentences

We observed that main finding sentences often have distinctive phrases, such as ‘we present’, ‘we report’, ‘to report’ and so on. Thus, we analyzed the presence of distinctive phrases as a distinguishing feature. This feature comprises three subtypes of textual patterns that are more frequently found in main finding sentences than other sentences. The first one is regular expression based, the second is syntactic based and the third is based on the first three words of the sentences. All items in all sub-patterns are displayed in Table S2.

To select these patterns, we extracted them from the set of positive vs. negative examples, and chose those that showed significant differences in frequency (i.e. a positive/negative frequency ratio > 2).

(2) \begin{equation*} \mathrm{discriminative}\ \mathrm{ratio}=\frac{I_{mf}/{N}_{mf}}{I_{os}/{N}_{os}}. \end{equation*}

Among them, ${I}_{mf}$ represents the number of main finding sentences containing this item; ${N}_{mf}$ represents the total number of main finding sentences; ${I}_{os}$ represents the number of other sentences containing this item; ${N}_{os}$ represents the total number of other sentences. Since these patterns may appear anywhere inside the sentence, the pattern strings were converted into lowercase to process pattern retrieval.

##### String-based patterns

We extracted word patterns which appear in >10% of main finding sentences in the manual corpus. These strings consist of single words and particular regular expressions. For example, ‘we * case’ is one of the particular pattern strings, that begins with ‘we’ and ends with ‘case’, while ignoring whatever words are in the middle. For retrieving kinds of strings, we use function ‘search()’ in Python. After filtering by discriminative ratio, there were six items in this subpattern.

##### Syntactic-based patterns

**Figure 2:**
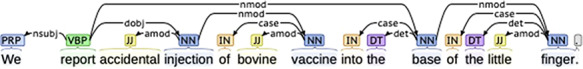
Example: basic dependencies of main finding.

We parsed all main findings and other sentences, by applying the dependency parser in Stanford Parser (version 3.9.2) tool, which is supported by the application programming interface of NLTK in Python 2.7 and Java Archive 1.8. The dependency parser gives a part-of-speech (POS) tag to each word in a sentence according to the relationship of all words. Figure 2 shows an example of basic dependencies of one main finding ‘We report accidental injection of bovine vaccine into the base of the little finger.’ The dependency tree presents POS tags of all words, as well as the dependency relationship between them and syntactic structure of the main finding.

After parsing, we scanned for the items ‘we+verb’, that is, a word ‘we’ with POS tag ‘PRP’ and its associated verb word with POS tag starting with ‘VB’. This pattern could occur anywhere inside the sentence, and there may be any number of words between ‘we’ and ‘verb’. After filtering by discriminative ratio, there were 15 items in this subpattern.

##### First 3-gram-based pattern

This pattern focuses on the first three words of the sentences. The most common first 3-grams that distinguish main finding sentences from other sentences were selected. After filtering by discriminative ratio, there were 37 items in this subpattern.

##### Scoring

The feature score of each item in each subpattern equals its discriminative ratio. For each sentence, we detect all the items in it and add up all the discriminative ratios of those items as the final score of that sentence. For example, sentence ‘To report a case of OFCD associated with a de novo BCOR pathogenic variant and highlight the ocular findings and possible mechanisms.’, it contains ‘to report a’ that fits one of the first 3-gram-based pattern with ratio 22, contains ‘report’ that fits one of the string-based patterns with ratio 6.19, contains ‘case’ that also fits one of the string-based patterns with ratio 4.15. So the sentence gets score = 22 + 6.19 + 4.15 = 32.34.

#### Feature 4. Words found more or less frequently in main finding sentences than in other sentences

In our study, we also explored how individual words are used differentially in main finding sentences vs. other sentences. In this feature, we extracted individual words whose frequency in main finding sentences were significantly higher or lower than in other sentences, without any stemming, lemmatization or stop word list being applied. The list was filtered to include only those words that occurred at least 25 times in the negative set; also, we removed those words that already appeared in the frequent textual patterns feature (Feature 3, above). Finally, we separated the words list into a positive words list consisting of words with discriminative ratios >2 and a negative words list consisting of words with discriminative ratios <0.5. Of the 109 words in the list, 49 were positive words and 60 were negative words. To calculate an overall feature score, we scanned all the words in one sentence; if one word appeared in the positive words list, the score of the sentence would be incremented by the discriminative ratio of that word; if the word appeared in negative words list, the score of the sentence would be subtracted by (1/discriminative ratio) of that word. If a word was expressed several times in the same sentence, it was counted only once. Below is the scoring formula.

(3) \begin{equation*} \mathrm{Pattern}\ \mathrm{Score}=\sum_{i=0}^n{ratio}_n-\sum_{i=0}^m{ratio}_m. \end{equation*}

*n* is the number of positive word in one sentences, and ${ratio}_n$ is the discriminative ratio of *n*th positive word; *m* is the number of negative word in one sentences, and ${ratio}_m$ is the corresponding ratio of *m*th negative word. All items in Feature 4 are shown in Table S3. A scoring example for sentence ‘Following a description about the characteristics of akinetic mutism (AM) and how it differs from locked-in syndrome (LIS) and a disorder of consciousness (DOC), we present the case of David, a 71-year-old man with AM.’, that contains ‘with’ in this feature with positive score 2.17, contains ‘syndrome’ in this feature with positive score 3.36, contains ‘a’ in this feature with positive score 2.39, contains ‘man’ in this feature with positive score 3.24, contains ‘it’ in this feature with negative score 2.71, so the final score of this sentence = 2.17 + 3.36 + 2.39 + 3.24–2.71 = 8.45.

#### Feature 5. Sentence location within the abstract

Since the main finding is the reason for writing up the case report, one might expect that it would be stated preferentially at the beginning or the end of the abstract. We tabulated the locations of main finding sentences vs. other sentences in unstructured and structured abstracts separately. For unstructured abstracts, we counted the number of times that a main finding sentence appeared in first sentence, second sentence, last sentence, second to last sentence and all other (‘middle’) sentences. Note that the locations were not partitions: for example, if an abstract consisted of only one sentence, this sentence was tabulated as being both the first and the last sentence. For structured abstracts, we counted the number of times that a main finding sentence appeared in the first section, second section, last section, second to last section and middle sections. In some cases of sentences belonging to more than one location (e.g. an abstract only consists of one sentence), we only allocate one position to those sentences with priority ranking: first, second, last, second to last and middle. According to formulas (2): discriminative ratio = $\frac{I_{mf}/{N}_{mf}}{I_{os}/{N}_{os}}$, we calculate discriminative ratios of each position for unstructured abstracts and structured abstracts, respectively. For unstructured abstracts, the discriminative ratios were 1.50 (first sentence), 1.08 (second sentence), 0.68 (middle sentences), 1.12 (second to last sentence) and 1.61 (last sentence). For structured abstracts, the discriminative ratios were 2.21 (first section), 0.69 (second section), 0.18 (middle sections), 1.97 (second to last section) and 2.21 (last section). Since the discriminative ratios of unstructured abstracts and structured abstracts presented similar distributions, we merged them and used ‘first’, ‘second’, ‘middle’, ‘second to last’ and ‘last’ to annotate this feature for all sentences in both types of abstracts, and finally transformed them into dummy variables with five categories.

**Table 1:** AUC values of individual features

Feature name	AUC value
Feature 1. N-gram-based similarity	0.86
Feature 2. Semantic similarity	0.79
Feature 3. Frequent patterns	0.82
Feature 4. Word frequency	0.85

#### Characterization of features by AUC values

In order to measure the ability of distinguishing main finding sentences vs. other sentences, we calculated area under curve (AUC) values for individual features. A value of 0.5 is no better than chance, whereas a value of 1.0 shows perfect ranking in which positive examples have higher scores than the negative examples. Table 1 shows the AUC values of features 1 to 4 (feature 5, being categorical, does not lend itself to this measure). Each individual feature has AUC > 0.8, indicating that it has substantial value for discriminating positive from negative sentences.

#### Correlation among individual feature scores

Except for Feature 1 vs. Feature 2, which measure title/abstract similarity in two different ways, the nonparametric Spearman rank correlations between feature scores are well below 0.5, indicating that the features measure substantially different attributes (Table 2).

**Table 2:** Correlations among individual features

Feature	Feature	Rank correlation
Feature 1. N-gram-based similarity	Feature 2. Semantic similarity	0.66
Feature 1. N-gram-based similarity	Feature 3. Frequent patterns	0.37
Feature 1. N-gram-based similarity	Feature 4. Word frequency	0.37
Feature 2. Semantic similarity	Feature 3. Frequent patterns	0.28
Feature 2. Semantic similarity	Feature 4. Word frequency	0.36
Feature 3. Frequent patterns	Feature 4. Word frequency	0.41

**Table 3:** Machine learning methods and parameters

ML method	Parameter
Support vector classification (linear kernel)	C = 1.0, kernel = ‘rbf’, degree = 3, gamma = ‘auto_deprecated’, coef0 = 0.0, shrinking = True, probability = True, tol = 0.001, cache_size = 200, class_weight = None, verbose = False, max_iter = −1, decision_function_shape = ‘ovr’, random_state = None
Support vector classification	C = 1.0, kernel = ‘rbf’, degree = 3, gamma = ‘auto_deprecated’, coef0 = 0.0, shrinking = True, probability = True, tol = 0.001, cache_size = 200, class_weight = None, verbose = False, max_iter = −1, decision_function_shape = ovr’, random_state = None
Nu-support vector classification	nu = 0.5, kernel = ‘rbf’, degree = 3, gamma = ‘auto_deprecated’, coef0 = 0.0, shrinking = True, probability = True, tol = 0.001, cache_size = 200, class_weight = None, verbose = False, max_iter = −1, decision_function_shape = ‘ovr’, random_state = None
Logistic regression	penalty = ‘l2’, dual = False, tol = 0.0001, C = 1.0, fit_intercept = True, intercept_scaling = 1, class_weight = None, random_state = None, solver = ‘warn’, max_iter = 100, multi_class = ‘warn’, verbose = 0, warm_start = False, n_jobs = None, l1_ratio = None
Multi-layer perceptron classifier (150 hidden layers)	hidden_layer_sizes = (150,), activation = ‘relu’, solver = ‘adam’, alpha = 0.0001, batch_size = ‘auto’, learning_rate = ‘constant’, learning_rate_init = 0.001, power_t = 0.5, max_iter = 200, shuffle = True, random_state = None, tol = 0.0001, verbose = False, warm_start = False, momentum = 0.9, nesterovs_momentum = True, early_stopping = False, validation_fraction = 0.1, beta_1 = 0.9, beta_2 = 0.999, epsilon = 1e-08, n_iter_no_change = 10
Random Forest Classifier	criterion = ‘gini’, splitter = ‘best’, max_depth = None, min_samples_split = 2, min_samples_leaf = 1, min_weight_fraction_leaf = 0.0, max_features = None, random_state = None, max_leaf_nodes = None, min_impurity_decrease = 0.0, min_impurity_split = None, class_weight = None, presort = ‘deprecated’, ccp_alpha = 0.0

### Machine learning

#### (a) Normalization of feature scores

In order to scale raw scores to an interval [0.0, 1.0], a linear normalization method was used to normalize word frequency-based feature scores:

(5) \begin{equation*} Nscore=\frac{Score- Smin}{Smax- Smin}. \end{equation*}

Among them, ‘Nscore’ represents the final normalization score; ‘Score’ represents the raw score; ‘Smax’ is the maximum score of the feature; and ‘Smin’ is the minimal score of the feature.

We used a log function-based algorithm to normalize N-gram-based similarity feature scores and frequent patterns-based feature scores.

**Log Normalization Algorithm:**

$$\begin{align*} \boxed{\begin{aligned} & if\ factor < 0\!: factor \leftarrow\ -1.0\\[-5pt] & else\ factor\leftarrow 1.0\\[-5pt] &\textrm{values}\leftarrow[\textrm{factor}^{\ast}\textrm{float}(\textrm{r}[\textrm{raw value}])\ \textrm{for}\ \textrm{r}\ \textrm{in rows}]\\[-5pt] &vmax\leftarrow max(values)\\[-5pt] & k\leftarrow 1.0/\ log(vmax\ +\ 1.0)\\[-5pt] & for\ r\ in\ rows\!:\\[-5pt] & r[normalization\ value]\!=\!\!k^{\ast}\!log(factor^{\ast}\!float(r[src\_key])\!+\!\!1.0) \end{aligned}} \end{align*}$$

Among them, ‘factor’ represents the positive or negative value of raw score; ‘values’ are the processed raw scores with negative and float attributes; and ‘vmax’ represents the maximum value among raw scores. In training and validation process, different sets of scores would have different maximum values; in other words, if the trained model were to be applied in a new dataset, ‘vmax’ is the maximum value among the new raw scores.

#### (b) Model construction and evaluation

Our scheme comprises two models. One model estimates the probability that a given sentence expresses a main finding. The second is abstract-level assessment of all of the sentences in an abstract, to identify which (if any) represent the best main finding sentence(s). In abstract-level assessment, the prediction may be one, more than one or no main finding sentence in that abstract.

### Sentence-level prediction

#### Sentence-level prediction model validation

In order to find the optimal combination of training features and build a quality main finding recognition model, we explored several different machine learning methods to train and validate our model. In the training process, we used cross-validation as follows: half of the data were randomly selected as training set and the remainder as test set, and this procedure was repeated 10 times, finally averaging the results.

#### Machine learning methods

Six machine learning methods were implemented by using a Python module called ‘Scikit-learn’ version 0.20.2 (13). Table 3 shows the six methods and their chosen parameters.

To evaluate internal performance of the machine learning methods, we applied 10-fold cross-validation, which randomly separates the positive and negative training examples into training set (90% of the data) and test set (10% of the data). For each sentence, the model produces a number between 0 and 1 that estimates the probability that the sentence states a main finding. This is converted into a binary yes/no prediction that is evaluated against training data.

### Abstract-level prediction

In reality, some abstracts contain no main finding sentences and some contain more than one. To assess the abstract-level performance of the model, the input to the model is an entire abstract, not a single sentence, and the output of the model is a prediction of which sentence(s), if any, are most likely to state the main finding. If the prediction score of a sentence is higher than an upper threshold (set at 0.9), we automatically give a positive label to that sentence; if the prediction score is below a lower threshold (set at 0.1), we automatically give a negative label. For those sentences whose prediction scores fall between 0.1 and 0.9, we followed two rules: (i) if one or more main finding sentence(s) were already identified in the first step, no other sentences are predicted as main finding; (ii) otherwise, the abstract sentence which has the highest prediction score is predicted to be the main finding. If the predicted positive sentence is the actual positive sentence, we marked this as ‘true positive’, and accuracy of abstract-level is defined as the proportion of true positive over the whole number of abstracts.

In order to prove these two thresholds are the optimal to the best of our work, we tested four different thresholds, which are lower or higher than the thresholds in our paper. Because there is no relationship between the low threshold and the high threshold, we used a lower threshold of 0.05 and a higher threshold of 0.2 for comparison of original threshold of 0.1; and a lower threshold of 0.8 and a higher threshold of 0.95 for comparison of original threshold of 0.9. Experimental results showed that all of the four changed thresholds would lead declined for the overall performance. We believe that the thresholds we selected are the optimal for our model.

**Table:** 

Support vector classification	Accuracy	Precision	Recall	F1
Threshold in our paper (0.1; 0.9)	**0.932**	**0.700**	**0.760**	**0.727**
Testing1 (0.2; 0.9)	0.926	0.722	0.663	0.691
Testing2 (0.05;0.9)	0.921	0.700	0.739	0.702
Testing3 (0.1; 0.95)	0.925	0.693	0.712	0.702
Testing4 (0.1; 0.8)	0.925	0.685	0.734	0.709

Boldface indicates the value(s) that has the best performance, and would be understood as such by most readers in this field.

In the manual corpus used for training, all titles either directly expressed or alluded to the main finding. However, in the biomedical literature as a whole, occasional case report articles exist in which the title has no relationship with the main finding. This would negate the value of features in our model that are based on measuring title-to-sentence similarity. If the highest semantic similarity between the title and any sentence in an abstract was <0.15, we implemented an alternative 3-feature model for abstract-level prediction that removed the title/sentence similarity features.

### (c) A new manual corpus of main finding sentences

In order to further evaluate the robustness of our model, we tested its performance on a newly created manual corpus of main finding sentences. We retrieved the PubMed identifiers (PMIDs) of clinical case reports (i.e. articles indexed as ‘case reports’ [Publication Type]) which were published from 1 January 2018 to 31 December 2018 inclusive and written in English (or with English abstracts). Of the articles retrieved, 200 were chosen at random using a random number generator. Two experienced annotators each examined these 200 case reports, and identified sentences that represented statements of main finding in title and abstract, using the guidelines previously described and carrying out reconciliation [55]. Note that this new manual corpus is more diverse than the one used for training the model, insofar as not all titles or abstracts necessarily contained a statement of the main finding, and not all had full text available in PubMed Central.

The 200 case report records were downloaded from PubMed in .xml format; PMID, title and abstract text were extracted and sentence tokenization was processed using the NLTK tool. The 200 abstracts consisted of 1480 sentences. A few parsing errors occurred: For example, NLTK is not able to recognize full stop followed by ‘•’ or full stop without a following space; as well, article keywords and other unrelated text embedded in the abstract field were recognized as sentences. We removed these problematic sentences from our evaluation, leaving 1474 sentences.

In the new corpus, 98.5% of the titles expressed the main finding. There are 29 abstracts lacking a main finding sentence, and 5 abstracts containing two adjacent sentences that state one main finding. In manual annotation, there were 176 main finding sentences, 11 alternative main finding sentences and 1287 other sentences.

## Results

The basic model estimates that probability that any given sentence states a main finding. Thus, in sentence-level evaluation, each sentence is scored, predicted and evaluated, so that performance reflects total predictions (and errors) calculated over the total number of sentences. Both annotated main findings and annotated ‘alternative main findings’ in the manual corpora are regarded as positive examples. In contrast, a more realistic use case for the model is to predict which sentence(s) within an abstract are most likely to state the main finding. Abstract-level predictions are calculated over the total number of abstracts. Abstract-level performance is likely to be lower than sentence-level performance because the situations are more complex (abstracts may state no, one or more than one main finding sentences).

### Sentence-level prediction

We used accuracy, precision, recall and F1 for measurement. Among them, sentence-level accuracy is defined as follows:

(6) \begin{equation*} \mathrm{Sentence}-\mathrm{level}\ \mathrm{accuracy}=\left({T}_p+{T}_n\right)/\mathrm{N}. \end{equation*}

Among them, ${T}_p$ represents number of true positive sentences, ${T}_n$ represents number of true negative sentences, N represents the total number of sentences.

**Table 4:** Model validation performance assessed by cross-validation of training data

ML method	AUC	Accuracy	Precision	Recall	F1
Support vector classification (linear kernel)	0.948	0.942	0.823	0.728	0.773
Support vector classification	0.943	0.935	0.794	0.702	0.745
Nu-support vector classification	0.949	0.942	0.820	0.729	0.772
Logistic regression	0.951	0.942	0.825	0.731	0.775
Multi-layer perceptron classifier (150 hidden layers)	0.947	0.941	0.822	0.726	0.771
Random forest classifier	0.915	0.929	0.748	0.735	0.741

**Table 5:** Sentence-level performance of the model on new test data (200 article manual corpus)

Baseline method		Accuracy	Precision	Recall	F1
Always the first sentence		0.787	0.147	0.166	0.156
Always the last sentence		0.814	0.249	0.28	0.263
Random sentence		0.801	0.198	0.223	0.210
Longest sentence		0.809	0.234	0.269	0.250
Share most title words (n-gram similarity)		0.882	0.505	0.568	0.535
ML method	AUC	Accuracy	Precision	Recall	F1
Support vector classification (linear kernel)	0.901	0.926	0.667	0.754	0.708
Support vector classification	0.904	0.932	0.700	0.76	0.727
Nu-support vector classification	0.900	0.931	0.700	0.756	0.725
Logistic regression	0.908	0.908	0.593	0.770	0.670
Multi-layer perceptron classifier (150 hidden layers)	0.899	0.920	0.645	0.733	0.686
Random forest classifier	0.847	0.885	0.506	0.682	0.581

Precision is defined as follows:

(7) \begin{equation*} \mathrm{Precision}={T}_p/\left({T}_p+{F}_p\right). \end{equation*}

Among them, ${T}_p$ represents number of true positive sentences, ${F}_p$ represents number of false positive sentences.

Recall is defined as follows:

(8) \begin{equation*} \mathrm{Recall}={T}_p/\left({T}_p+{F}_n\right). \end{equation*}

Among, ${T}_p$ represents number of true positive sentences, ${F}_n$ represents number of false negative sentences.

F1 is defined based on precision and recall as follows:

(9) \begin{equation*} \mathrm{F}1=2^\ast \mathrm{precision}^ \ast \mathrm{recall}/\left(\mathrm{precision}+\mathrm{recall}\right). \end{equation*}

As shown in Table 4, the sentence-level model performed well as assessed by 10-fold cross-validation on the positive vs. negative sentences taken from the 416-article manual corpus used for training. The AUC and F1 did not vary markedly across different machine learning methods tested.

When the trained model was tested on sentences taken from a new manual corpus of 200 randomly chosen case reports (see Materials and Methods), the results on new data are a little lower than in Table 4, but AUC and accuracy values are still high (Table 5). To the best of our knowledge, this is the first study for identification of main finding in case reports. In order to assess our model’s effectiveness, we compared it against a variety of baseline methods: (i) always choose the first sentence as main finding, (ii) always choose the last sentence as main finding, (iii) choose the main finding sentence at random, (iv) always choose the longest sentence as main finding and (v) choose the sentence with highest n-gram similarity with title. As shown in Tables 5 and 6, all baselines gave markedly worse performance than our optimized model.

**Table 6:** Abstract-level performance on new test data (200 article manual corpus)

Baseline method	Abstract-level accuracy
Always the first sentence	0.161
Always the last sentence	0.276
Random sentence	0.218
Longest sentence	0.264
Share most title words (n-gram similarity)	0.563
ML method	Abstract-level accuracy
Support vector classification (linear kernel)	0.64
Support vector classification	0.66
Nu-support vector classification	0.66
Logistic regression	0.64
Multi-layer perceptron classifier (150 hidden layers)	0.625
Random forest classifier	0.58

We define abstract level accuracy as follows:

(10) \begin{equation*} \mathrm{abstract}-\mathrm{level}\ \mathrm{accuracy}={T}_a/{N}_a. \end{equation*}

Among them, ${T}_a$ represents the number of abstracts that the model has predicted the true main finding as positive sentence, ${N}_a$ represents the total number of abstracts.

The accuracy of abstract-level prediction results when tested on the 200 case reports corpus showed an apparent drop in performance compared with the sentence-level prediction (see Table 6 vs. Table 5).

One difference between the manual corpus used for training, and the new manual corpus used for testing, is that all abstracts in the training set had exactly one main finding sentence, whereas some abstracts in the new corpus contained no, one or two main finding sentences. To examine whether this accounted for the drop in performance on new data, we computed sentence-level (Table 7) and abstract-level (Table 8) performances for the subset of test data (175 articles) that contained one main finding sentence. Indeed, the sentence-level performance on test data is as good as on the training data. Abstract-level accuracy is also much improved (Table 8 vs. Table 6).

**Table 7:** Sentence-level prediction on new test data whose abstracts contained one main finding sentence

ML method	AUC	Accuracy	Precision	Recall	F1
Support vector classification (linear kernel)	0.907	0.934	0.756	0.751	0.754
Support vector classification	0.910	0.939	0.780	0.757	0.769
Nu-support vector classification	0.906	0.938	0.780	0.753	0.766
Logistic regression	0.913	0.919	0.680	0.767	0.721
Multi-layer perceptron classifier (150 hidden layers)	0.904	0.927	0.729	0.729	0.729
Random forest classifier	0.852	0.891	0.574	0.677	0.621

**Table 8:** Abstract-level prediction on new test data whose abstracts contained one main finding sentence

ML method	Abstract-level accuracy
Support vector classification (linear kernel)	0.758
Support vector classification	0.764
Nu-support vector classification	0.764
Logistic regression	0.770
Multi-layer perceptron classifier (150 hidden layers)	0.739
Random forest classifier	0.679

## Error analysis

We chose the evaluation results from support vector classification for error analysis, since it gave the best performance in our model. From the sentence-level aspect, 100 (out of a total of 1474) sentences got prediction labels that were inconsistent with the manual corpus labels. Among them, 27 predictions are arguably not true or frank errors as judged by post-hoc scrutiny: 17 of them were evaluated post-hoc as acceptable or even better main findings than the one marked in the manual corpus and 6 were cases in which the predicted sentence was marked as an alternative main finding in the manual corpus. In the five abstracts in which two adjacent sentences formed one main finding, the first sentence was always predicted as the main finding, which can be regarded as a partial error (since only the second sentence is missed).

The remaining 72 errors are analyzed in Table 9. The most common error occurred when the predicted sentence states the case presentation, since both types of sentences may begin with pattern features such as ‘We present’. Among the 29 abstracts that lacked any annotated main finding, the model predicted a main finding in 22 of them. Rarely, no sentence in the abstract received a score > 0.1 and so no main finding was predicted.

**Table 9:** Error analysis of 72 errors

Reason	Number
Predicted sentence states the case presentation	28
Abstracts have no main finding, but model predicted one main finding sentence	22
Predicted sentence is wrong (main finding has lower score)	10
All sentences in the abstract have scores <0.1	5
Predicted sentence states the take home message	4
Predicted sentence states the background	2
Parsing error caused the predicted sentence to receive incorrect score	1

## Discussion

In the present report, we have created and evaluated a machine learning-based model to estimate the probability that a sentence within the abstract of a clinical case report states the main finding. Five features were employed that comprised word n-gram and semantic similarity to the title, words and patterns that are differentially expressed in main findings vs. other sentences and location of the sentence within the abstract. The training procedure utilized positive and negative examples from a previously created manual corpus (5), and we evaluated performance at both sentence-level and abstract-level on a newly created manual corpus (Supplemental File 1).

Our annotation and extraction of main finding sentences is generally related to other information extraction efforts that have looked for sentences that assert knowledge claims (10, 14–16), that are descriptions of clinical outcomes (17), that are classified into categories (18, 19), that summarize the article as a whole, or lists of topics, concepts or keywords that are discussed in the article. The present study is perhaps most similar to Shardlow *et al.* (9) who identified sentences that present New Knowledge (an author’s findings). This is similar to our idea insofar as the main finding of a case report represents a particular context for presenting New Knowledge. However, they applied their annotations to abstracts concerned with experimental studies of transcription factors and employed intra-sentence linguistic features for modeling. In contrast, since clinical case reports are much more likely to state the main finding directly in the title, we used the title as a probe to recognize the corresponding statement(s) of main finding within the abstract.

## Limitations

The current model is over-simplified in at least two respects. First, in the rare situations when the title of the case report did not have lexical or semantic similarity to the main finding at all (3 out of 200 articles in the new manual corpus), we were forced to apply a simpler 3-feature model. Second, the model distinguishes main finding sentences vs. all other sentences, without specifically modeling two types of sentences that can sometimes mimic the main finding—the sentence that presents the case and the sentence(s) that discuss implications of the main finding (i.e. the take home message).

In our previous description of manual corpus development, ‘Working independently, the annotators agreed on the abstract main finding in 322/500 = 64.4% of cases. Cohen’s kappa before discussions = 0.593 that represents “moderate agreement”’ (5). Our error analysis using the new manual corpus also found some articles where post-hoc evaluation disagreed with the annotators (even after they had reconciled). These findings suggest that one of the factors limiting our model’s ability to identify main finding sentences is the simple fact that they are not always well formed or easy to identify even by human readers.

## Future research enabled by the model

Why did we create a model to estimate the probability that a sentence states the main finding of a case report? As mentioned in the Introduction, we hypothesize that the present model will enable at least four lines of research.

(i) It would be desirable to index case report articles according to their main findings. In our preliminary studies, we have found that the title alone does not provide sufficient information for this purpose. Thus, we hypothesize that combining text judiciously from both title and main finding sentence(s) will provide additional information that will assist in indexing. We have also found that a typical case report contains sentences that state the main finding not only in the abstract, but repeated in a non-verbatim manner in one or more places within the full text (e.g. in the Introduction and Conclusions sections) (5). The multiple statements of main finding within a single case report will share certain common features, and each statement may add other valuable information or may add irrelevant ‘noise’. It will be an interesting challenge to see how the different main finding statements can be combined into a single composite statement that contains the maximal overall relevant information content while minimizing irrelevant information.
(ii) Identifying the main finding sentence of the same article is the first step in creating a similarity metric that can be applied to identify similar main findings across different articles. Retrieving case reports according to their main findings (rather than general topics) would allow users to find all reports that state the same (or closely related) main finding as a given case report. The case report literature is quite scattered and poorly cited, so that it is not easy to recognize when multiple reports state the same main finding. Such situations are important because, although any one report may be noisy and uncontrolled, the presence of multiple reports should greatly increase their overall confidence and credibility (2, 6–8).
(iii) Identifying main finding sentences and other information within other types of articles, e.g. clinical trial articles, is a more complex situation requiring deeper linguistic and argumentation mining (20, 21). Employing such techniques for case reports may not only improve predictive performance for main findings but may also help generalize information extraction across diverse types of articles.
(iv) Finally, the multiple different statements of the same main finding, in the same paper, can be viewed as multiple sentences that say ‘almost’ the same thing. This should provide a textual resource to supplement sentence similarity, paraphrases and natural language inference in biomedical text more generally (22, 23).

### Implementation

The code and necessary files to run the main finding model can be downloaded from https://github.com/qi29/main_finding_recognition, released under the Apache License, Version 2.0.

## Supplementary Material

Supplemental_File_1_Manual_Corpus_(1)_baaa041

Table_S1_baaa041

Table_S2_baaa041

Table_S3_baaa041

## Supplementary Data

Table S1 displays the stop word list applied in Feature 1 and Feature 2.

Table S2 shows the list of items scored in feature 3. Table S3 shows the list of items scored in Feature 4.

Supplemental File 1: This Excel spreadsheet displays the main finding sentences of 200 clinical case reports as annotated by two raters SA and NS (see Materials and Methods for description).
